# Interdisciplinary Medication Adherence Program: The Example of a University Community Pharmacy in Switzerland

**DOI:** 10.1155/2015/103546

**Published:** 2016-01-10

**Authors:** Mélanie Lelubre, Susan Kamal, Noëllie Genre, Jennifer Celio, Séverine Gorgerat, Denise Hugentobler Hampai, Aline Bourdin, Jerôme Berger, Olivier Bugnon, Marie Schneider

**Affiliations:** ^1^School of Pharmaceutical Sciences, University of Geneva-University of Lausanne, 1205 Geneva, Switzerland; ^2^Community Pharmacy, Department of Ambulatory Care & Community Medicine, University of Lausanne, 1011 Lausanne, Switzerland

## Abstract

The Community Pharmacy of the Department of Ambulatory Care and Community Medicine (Policlinique Médicale Universitaire, PMU), University of Lausanne, developed and implemented an interdisciplinary medication adherence program. The program aims to support and reinforce medication adherence through a multifactorial and interdisciplinary intervention. Motivational interviewing is combined with medication adherence electronic monitors (MEMS, Aardex MWV) and a report to patient, physician, nurse, and other pharmacists. This program has become a routine activity and was extended for use with all chronic diseases. From 2004 to 2014, there were 819 patient inclusions, and 268 patients were in follow-up in 2014. This paper aims to present the organization and program's context, statistical data, published research, and future perspectives.

## 1. Introduction

A widely known challenge in chronic healthcare is the percentage of patients who do not follow their medication regimen, from poor adherence to nonadherence [1]. When patients take their medications as prescribed, they are considered to be adherent. Adherence has two components that are complementary to each other: persistence and implementation. Persistence describes the length of time between the first and last doses. Implementation describes the initiation of treatment (when the patient takes his or her first dose); the implementation of the dosing regimen (if the patient follows the actual prescribed dosing regimen); and, finally, the discontinuation of treatment [2]. The World Health Organization (WHO) estimates that only 50% of chronic patients correctly take their medication, depending on the patient's disease, medication, and demographic characteristics [1]. For example, 62% of the world's HIV-positive population is more than 90% adherent to medication [3]. Nonadherence has obvious consequences not only for a patient's health (e.g., nonefficacy of the treatment or disease progression) but also for healthcare costs (e.g., the number of medical visits, rate of hospitalization, extended stays in hospitals, or multiplication of diagnostic tests) [4, 5]. According to a 2012 report by the IMS Institute for Healthcare Informatics, 8% of total healthcare costs worldwide are spent unnecessarily, due to the suboptimal use of medicines. Out of that 8%, approximately 57% is due to nonadherence, which represents $270 billion dollars [6]. In Switzerland, costs related to nonadherence are estimated to be 30 billion CHF, which is 50% of annual health care costs [7].

Addressing the problem of nonadherence would evidently help alleviate the burden of added costs to healthcare. To do so, healthcare systems need to change by developing interdisciplinary medication adherence intervention programs to ensure better adherence to medication, especially to chronic therapies [1]. Worldwide, there are some activities within healthcare systems that support or monitor medication adherence as part of more comprehensive programs, such as medication review (Australia, Spain, Denmark, and Finland); identification of drug-related problems (Sweden); and medication checks (Denmark) [8]. The pharmacist delivers the majority of these programs, but there are some that are multiprofessional, involving physicians or nurses. There are also initiatives that do not involve pharmacists. For example, the Cincinnati Children's Hospital Medical Center (USA) developed and implemented an empirically informed comprehensive model of pediatric adherence promotion in the management of pediatric chronic conditions [9]. Some governments start to reimburse pharmacists for related services to support medication adherence, such as in Switzerland, England, or Belgium [8, 10]. For example, in England, they introduced the third round of pharmaceutical services (or the New Medicine Service, NMS) in 2011 to promote the health and well-being of patients who start a new medication [11].

In 2004, based on previous experience with other chronic diseases, the Community Pharmacy of the Department of Ambulatory Care and Community Medicine (Policlinique Médicale Universitaire, PMU), University of Lausanne, in collaboration with the Infectious Diseases Service of the Lausanne University Hospital (CHUV, Lausanne, Switzerland), developed and implemented an interdisciplinary antiretroviral therapy (ART) adherence program for HIV patients. Since then, this program has become a routine activity and was extended for use with all chronic diseases. The program aims to support and reinforce medication adherence through a multifactorial and interdisciplinary intervention. According to the National Institute for Health and Care Excellence's (NICE) 2009 Guidelines, it is important to involve patients in decisions about prescribed medicines and adherence support [12]. This is why the pharmacist, who is part of the developed intervention program at the PMU, is interested in empowering the patient's autonomy with treatment as much as possible. The adherence program's main goal is to increase medication adherence to achieve therapeutic goals. However, it is important to set achievable, realistic intermediate subgoals to initiate and maintain patient-empowered long-term treatment behavior. Motivational interviewing is combined with medication adherence electronic monitors (MEMS, Aardex MWV, Switzerland) and report that provides feedback to the patient, physician, nurse, and other pharmacists. Electronic measure of medication adherence allows professionals to provide feedback to the patient on the dosing history and thereby enhance patient medication adherence [13].

This paper aims to describe the organization and program's context, statistical data, published research on medication adherence interventions, and future prospects for the medication adherence program at the PMU.

## 2. Methodology

### 2.1. Background Information

**Figure 1:**
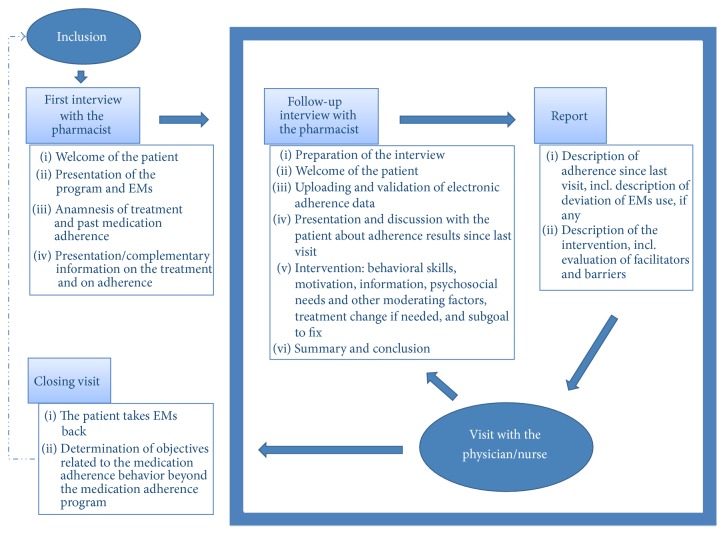
Medication adherence program—organizational process.* Note.* EMs = electronic monitors of medication adherence.

The physician, pharmacist, or nurse invites the patient to take part in the program, but in practice, the physician is often the best person to invite the patient to participate in the program because of the established patient-provider relationship. The first interview between the patient and pharmacist happens on the same day or within a few days after the patient agrees to participate in the program. The following interviews are arranged with the patient and called follow-up interviews, which take place 30–45 minutes before a medical visit and last for 15–30 minutes. The medication adherence interview is conducted when medicines are given and happens in an interview room to ensure confidentiality. At the beginning of each interview, the pharmacist validates the medical prescriptions to ensure treatment is prescribed in a safe, effective, adequate, and economic way and according to evidence-based medicine. At the end of each follow-up interview and with the patient's consent, a report is sent to the physician. The patient is informed that the pharmacist, physician, and nurse exchange information to support medication adherence. Any decision to stop the program is made mutually by the physician and pharmacist in collaboration with the patient. In this case, a discontinuation visit takes place with the pharmacist (see Figure 1).

The frequency of interviews depends first on the patient's needs and second on his or her availability (on average, once a month to once a trimester). For example, an HIV patient starting a new treatment attends a medical visit at 2 and 4 weeks and at 2, 3, 4, and 6 months if no problem of toxicity appears. The pharmacist organizes the medication adherence interview according to these medical visits. The pharmacist also plans extrainterviews in between medical visits, in case of difficulties to adherence that could potentially lead to clinical consequences. Special attention is given at the beginning of follow-up.

Medication adherence interviews are scheduled in the pharmacy's electronic agenda to keep track of all patients. A pharmacy technician, specialized in medication adherence, checks the agenda at least once a day and validates the daily list of scheduled patients. If the next interview date is not yet scheduled with a patient, the technician enters an electronic reminder to contact the patient 10 days before a shortage of medicines occurs. If a patient does not come to an interview, the technician contacts the patient to arrange another appointment. In the case of 3 unsuccessful calls and before a shortage of medicine, the pharmacist contacts the physician to decide on a mutual action. The physician invites the patient to return for a medical appointment and to visit the adherence clinic.

All information is collected electronically at the pharmacy, which uses 2 software programs for the medication adherence program: medAmigo [14] or SISPha [15].

#### 2.1.1. Inclusion of Patients

When a patient agrees to take part in the adherence program, the physician calls the pharmacist, who completes a document for the “medication adherence program request.” This document gathers details on the patient (name, sex, birth date, address, phone number, spoken languages, availability of a translator if needed, and half-day preferences for interviews); details on the physician (contact information and knowledge of the adherence program); and clinical data (reasons for the request, diagnosis and comorbidities, complete medicine treatment, and determination of medicines for monitoring).

The main reasons to include a patient in this program are the following:

- failure to achieve therapeutic goals;
- adherence difficulties exposed by the patient and/or discussed with the physician, pharmacist, or nurse;
- difficult psychosocioeconomic conditions;
- complexity of the current treatment (increasing dose or dosage, introduction of a new medicine);
- comorbidities known to affect medication adherence (e.g., depression, anxiety, illicit drug use, and alcohol);
- history of nonpersistence to treatment;
- frailty known to have an impact on medication adherence (e.g., teenagers, especially during their transition from pediatric to adult medicine, pregnancy, and postpartum in HIV+ women).

#### 2.1.2. First Interview with the Pharmacist

The interview consists of 4 parts. During the first part of the interview, the pharmacist describes the nature and direction of the program, which is completely new to most patients; sets the framework (time over which interviews take place, length of interviews); and assesses the patient's readiness to start or keep taking the treatment. While the pharmacist is the one who guides the interviews (semidirective intervention), the patient is invited to actively participate in the interviews, according to the principle of patient-centered care. The interviews can be attended by the patient alone or with a significant other. A 1-page leaflet with written information is provided to the patient. This document also attests that the electronic monitors (EMs) are delivered on a loan basis to the patient and must be returned at the end of the program. Otherwise, the pharmacy would claim the cost of the EM by sending an invoice to the patient. This document is then signed and dated by the patient and pharmacist.

During the second part of the interview, the pharmacist gets to know the patient's personal and psychosocial conditions (e.g., if the patient lives alone or is surrounded by a circle of social support); therapeutic history (current and previous treatments, doses and dosages, former nonadherent and nonpersistent behaviors, and reasons); experienced barriers and facilitators to medication intake; and previous adherence support, if any (e.g., pillbox, directly observed therapy, home nurse, significant-other support). Having this information then starts the intervention.

During the interview's third part, the pharmacist presents the EMs and verifies that the patient can adopt them without any major difficulties (e.g., manual dexterity or visual issues or neurocognitive troubles). The patient is informed that the EM records the day and the hour of each opening and that this information is made available to the patient at any time through the LCD screen on top of the monitor. To prevent a mix of monitors and their containers, each corresponding EM and its container are marked with the same color. The patient is asked to bring back EMs at each medication adherence interview, even if there is still some medicine remaining, as the pharmacist always delivers more medicine than necessary to anticipate any appointment changes.

During the interview's fourth part, the pharmacist presents the monitored treatment and provides medical pamphlets. Afterward, the patient and pharmacist schedule medication intake by combining the pharmaceutical recommendation with the patient's own preferences, and they define individual, appropriate use for the patient. In case of drug continuation, the pharmacist considers the patient's current and previous organization, as well as capturing former and actual barriers and facilitators to support the patient in finding sound, individual, and appropriate use.

Finally, the pharmacist invites the patient to ask questions, summarizes the interview, and eventually sets a realistic short-term goal to meet first for the next interview and fixes the next appointment.

#### 2.1.3. Follow-Up Interviews

Follow-up interviews focus on the patient's adherence and medicine-related issues (e.g., regimen, side effects). The pharmacist prepares the interview and at least reviews 2 to 3 previous reports, including the inclusion document if he or she does not know the patient.

The pharmacy technician uploads EMs data and counts returned pills. The differences between electronic adherence and pill count exceeding 20% are explored afterward. At the start of the interview, the pharmacist may remind the patient about the nature and direction of the program and provide a summary of the last interview. Before presenting the latest data on the EMs, the pharmacist validates the electronic data and assesses self-reported adherence by asking the patient the following questions:

- Within which time interval do you take your medicine after opening the EMs? The answer is then categorized (≤1 hour, >1 hour, and variable).
- Are EMs consistently used for each dose or did you organize pocket dosing or experience nonmonitored periods (e.g., during a hospitalization where treatment was delivered from the hospital supply by the nursing staff)? The pharmacist notes dates and reasons for each deviation of EM use with the greatest possible accuracy.
- Do you think you have missed taking some pills since the last interview? If yes, then the patient describes when, how much, and in which circumstances and reasons (unintentional/intentional). This information allows the pharmacist to identify whether self-reported adherence is close or not to electronic adherence and guide the presentation of EM results by accounting for the patient's attitude.

**Figure 2:**
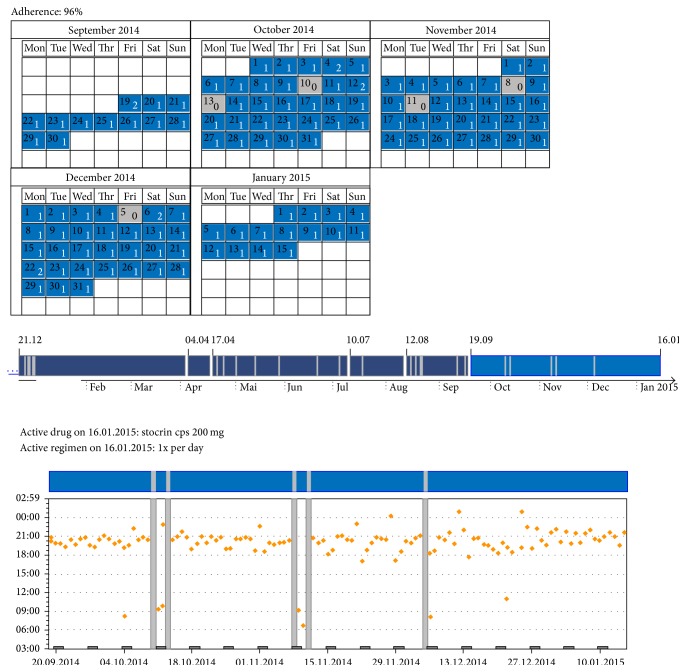
Example: results of an electronic monitor—calendar and graph (from medAmigo [14]).

After validating the use of the EMs, the pharmacist shows, describes, and discusses the EM results with the patient. The results are displayed using patient-friendly graphs (calendar and chronology, see Figure 2). Showing the report often generates spontaneous patient comments and steers the discussion. The pharmacist congratulates the patient on the days with correct medication intake and encourages small progress. If the EM depicts missed doses, the pharmacist pays attention to the most recently missed ones and then goes back in time and asks the patient to describe what happened that was usual or unusual on the same day. The patient exposes perceived medication intake barriers and facilitators.

**Figure 3:**
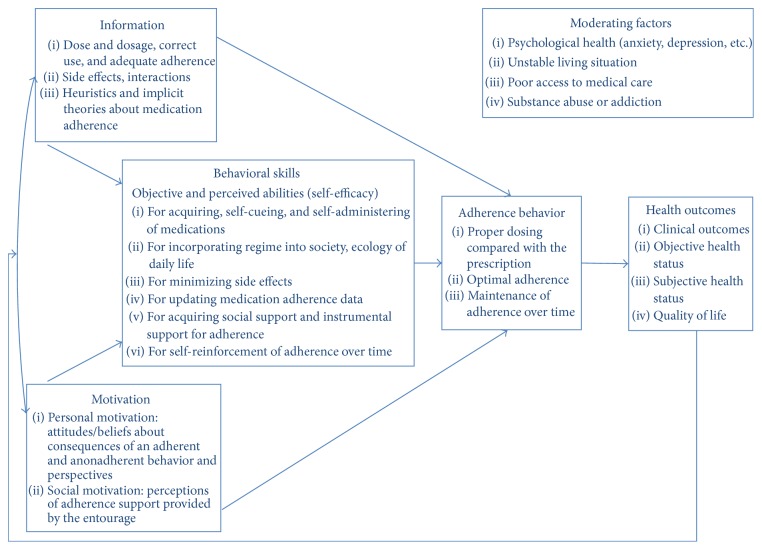
Fisher's model—Information-Motivation-Behavioral Skills model of adherence to antiretroviral treatment (adapted from Fisher et al. [16]).* Note.* HAART = highly active antiretroviral therapy.

The intervention's framework is based on the sociocognitive theory, especially the Information-Motivation-Behavioral Skills model (IMB model, see Figure 3) [16]. This comprehensive, cognitive, motivational, and behavioral intervention progresses at the patient's rhythm, using motivational interviewing skills by paying particular attention to the patient's language of change, thanks to empathy and active and reflective listening. During the interview, nonadherent patients are invited to describe their attitude and barriers to medication intake. At the same time, the pharmacist helps them in exploring facilitators to medication intake, and this eventually results in the adjustment of their own behavior in a timely and autonomous way.

The cardinal points of the intervention are as follows:

- *Information.* The pharmacist assesses the patient's knowledge (adherence, treatment, and disease); informs him/her according to individual, evolving needs; and/or refers the patient to the physician.
- *Motivation in Medication Intake.* The pharmacist assesses the patient's motivation; patient's own clinical and quality of life expectations; and emotions linked to the treatment (e.g., anxiety, fear, disinterest, and denial) to check that emotional needs are balanced, such as by expectations and/or social or practical support that the patient receives.
- *Behavioral Skills.* The pharmacist explores the levels of integration of medication intake in the patient's daily routine; self-efficacy; capacity to self-manage treatment and side effects; and the patient's strategies to prevent missed doses (e.g., management at home and if out of the home, during weekends, holidays, or in case of an unfamiliar schedule, reminders, and storage).

In case the physician foresees a change in treatment, the pharmacist explores the patient's opinion about this change and discusses previous change experiences, if any, and the patient's readiness for this upcoming change.

Moderators of Fisher's model—such as psychological clinical or subclinical deficit, unstable living situation, poor access to medical care, and substance abuse—are considered. According to the identified variable affecting adherence, the pharmacist, physician, or nurse takes the lead in supporting the patient. For example, in case psychosocial components affect adherence, the patient could be referred to a psychologist or psychosocial worker. In case treatment complexity is the main trigger of nonadherence, the pharmacist and the physician identify ways to simplify treatment. Finally, the pharmacist helps the patient in setting realistic goals until the next interview and summarizes the interview. Then, the pharmacist fixes the next appointment and invites the patient to call him/her in case of intercurrent medication intake problems.

The pharmacist finalizes the medication adherence report and sends it to the physician at a maximum of 3 days after the interview or gives the report directly to the patient if the medical visit happens on the same day. The three main goals of this electronic report are to ensure (1) continuity of care between the pharmacist, physician, and nurse and a multidirectional flow of information; (2) continuity of care at the pharmacy between interviews, with the possibility to easily notice changes in patient adherence (maintenance, increase, and decrease over time); and (3) quality management and activity's traceability. The report presents the EMs' adherence results and the pharmacist's comments according to 3 sections:

- Objective description of the patient's adherence since the last interview and validation of EMs use (overall adherence and timing, description of days without intake—isolated, clustered, change over time—with a focus on special life events, which affect adherence).
- Description of medication adherence facilitators and barriers encountered by the patient, including medication side effects and symptoms.
- Summary of the intervention based on the IMB model and adherence subgoal(s) for the next interview.

After each medical visit, the patient returns to the pharmacy to fill the EMs with the prescribed pills until the next appointment.

#### 2.1.4. Phases and Closing the Medication Adherence Program

The medication adherence program is divided into 3 phases:

1. *Initiation Phase (Generally Months 0 to 6).* Problems are clearly identified and a search for solutions takes place. During this phase, the patient is followed as often as possible by the same pharmacist (in collaboration with a substitute pharmacist).
2. *Consolidation Phase (Generally Months 6 to 12).* The patient enters this phase if he or she achieved 4 conditions: (1) he or she achieved or came significantly closer to his or her therapeutic goals, (2) medication adherence improved and has been adequate for a minimum of 4 months, (3) identified barriers to adherence are either resolved or balanced by facilitators, and (4) at least 3 adherence interviews were conducted. During this phase, the patient consolidates the newly learned adherence behavior, and it is no longer necessary to be seen by the same pharmacist every time, as in the previous phase.
3. *Maintenance Phase (Generally Months 12 and Later).* Adherence has been adequate for the last 12 months, barriers encountered are solved or self-managed by the patient, and a major clinical improvement in therapeutic goals is achieved. Patients are invited to stop the program after the pharmacist, physician, and nurse agree on it. However, some patients express their wish to continue the program. This wish can be because the EMs structure the patient's daily medication intake, and the electronic feedback given during medication interviews increases the patient's feeling of security. In this case, uploads of EMs data are scheduled 3 to 4 times a year, and interviews are short, based on electronic feedback. If new adherence issues emerge, the patient is invited to enter a new initial phase.

At the end of the program or if the patient decides to quit prematurely, a closing visit is scheduled. The reason for closing is detailed in the report, especially whether treatment is pursued or not. The pharmacist informs the patient of the possibility of relapse and ways to decrease its risk, including letting the patient know that the adherence program remains available at any time.

#### 2.1.5. Handling EMs

To maximize efficiency, pharmacy technicians are in charge of logistics and technical work linked to EM management. They activate and program EMs and check their functional use before the first delivery and also upload EM data before each interview. The pharmacists prepare and fill in EMs at each prescription refill and document this activity in a dedicated form. Additionally, the pharmacist verifies the medicine stability in EMs.

### 2.2. Training the Team

The medication adherence program is ISO certified. All procedures are written and made available to the team at any time.

#### 2.2.1. Pharmacists' Training

First, the new pharmacists receive basic training. They attend a 10-hour class organized into 5 modules: (1) introduction to medication adherence, (2) theoretical frameworks of medication adherence, (3) medication adherence intervention programs, (4) structure of medication adherence interviews, and (5) case studies. After these modules, pharmacists in training observe a minimum of 3 medication-adherence interviews with a trained pharmacist.

Second, pharmacists are trained in motivational interviewing during four 4-hour sessions [17]. They include practical exercises, discussions, illustrations, and role play. Then, participants have the opportunity to benefit from a filmed interview with a simulated patient and receive feedback from a professional.

Third, pharmacists in training deliver 3 to 8 medication-adherence interviews, depending on the pharmacist's experience and skills, under the supervision of a senior trainer (pharmacist) who attends the interview, gives feedback after each interview, and validates each medication adherence report.

To maintain a high and standardized quality level for the medication adherence program, one interview per pharmacist is recorded every 18 months, and a debriefing with a trainer is conducted. Every 6 to 8 weeks, a 1-hour adherence internal meeting is organized for educational purposes and discussing complex case studies.

#### 2.2.2. Pharmacy Technicians' Advising

Pharmacy technicians are advised on the spot by trained technicians and pharmacists. While there is no formal training for the new technicians, they participate in the adherence internal meetings.

## 3. Results and Discussion

### 3.1. Context and Published Research

The Community Pharmacy of the Department of Ambulatory Care and Community Medicine is linked to the Research Unit of Community Pharmacy, School of Pharmaceutical Sciences, University of Geneva-University of Lausanne. The Community Pharmacy practice helps and gives ideas to the research team and in parallel, this academic setting promotes research and development [18].

Progressive for the time, the medication adherence program started in 1995. The Community Pharmacy introduced EMs without showing medication adherence data to patients and physicians. When results were shown for the first time to patients, pharmacists and physicians noticed patients' positive reactions [19]. In 2004, a new interdisciplinary collaboration with the infectious diseases service of the CHUV boosted the program with the inclusion of HIV patients, so the program became a structured routine activity [20–24]. Interdisciplinary collaboration leads to a safer and coordinated healthcare system for patients and their families with more involvement in decision-making. It also facilitates access to healthcare interventions [25]. The number of trained pharmacists increased, and pharmacy technicians were integrated in the program. We did a hand search of all published literatures on the medication adherence program at the PMU. Details can be found in Supplementary Appendix  1 (see Supplementary Appendix  1 in the Supplementary Material available online at http://dx.doi.org/10.1155/2015/103546).

The intervention follows the Behavior Change Technique Taxonomy by Michie et al. that was developed to provide an extensive consensually agreed structured taxonomy for behavior change techniques used in interventions [26] (see Table 1).

### 3.2. Results Describing the Adherence Program Activity

**Figure 4:**
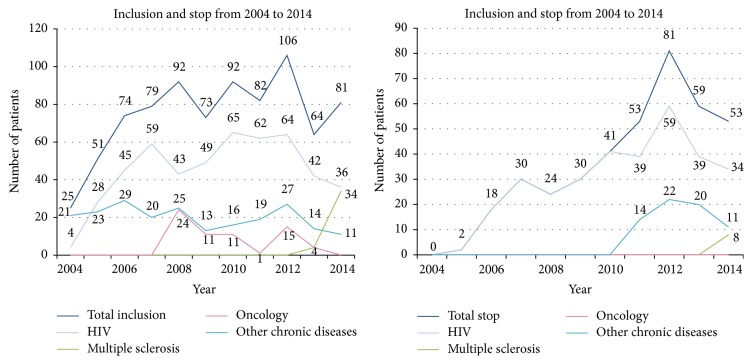
Inclusion in the medication adherence program and stop from 2004 to 2014.* Note.* Patients who reentered the program after a gap of more than 1 year were considered to be new inclusions.

**Figure 5:**
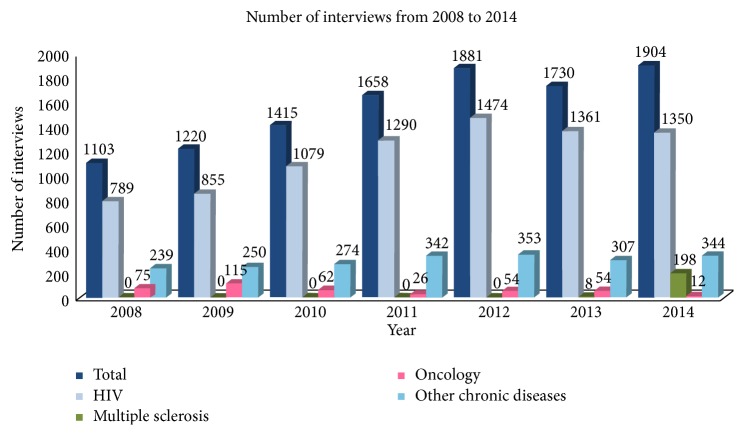
Number of medication adherence interviews delivered by pharmacists.

From 2004 to 2014, 819 patients were included and pharmacists delivered 10'911 interviews for different chronic diseases from 2008 to 2014 (see Figures 4 and 5). All patients accepted using EMs, and EMs are not imposed on patients; alternative solutions are available, for example, weekly pill organizers. In 2014, 268 patients were followed up, out of whom 187 were HIV patients; 28 multiple sclerosis patients; 9 oncology patients; and 44 patients for other chronic diseases (e.g., hypertension, type 2 diabetes, and chronic dialysis).

On average, patients have 2 EMs over their entire follow-up period [IQR: 1–3; min-max 1–8]. The duration of the follow-up is long with a median of 333 days for HIV patients [IQR: 138–799; min-max 11–3317]. Medication adherence is a dynamic process that needs long-term follow-up [1]. Indeed, such a longitudinal measure of patients' medication adherence allows for capturing adherence's evolution over time. For example, pharmacists in this program detect the precursory signs of a possible deterioration of medication adherence (e.g., increase in timing fluctuation of medication intake) and work with patients during the interview on preventing further deterioration. Two consequent interviews are generally separated by a median of 33 days [IQR: 15–77]. The median is very close for HIV, multiple sclerosis, and other chronic diseases but is higher for oncology with a median of 49 days [IQR: 30–95] (see Table 2).

To run this program, the pharmacy needs 1 full-time pharmacist, 1 full-time pharmacy technician, and 1 alternate pharmacist for busy days. On average, interviews last for approximately 10 minutes [IQR: 5–15]. Pharmacy technicians take 20 minutes [IQR: 15–25] to prepare medicines for the first interviews and 13 minutes [IQR: 9–20] for follow-up interviews, and pharmacists take 25 minutes [IQR: 17–36.5] to complete the report for the first interviews and 12 minutes [IQR: 7–20] for follow-up interviews (see Table 2).

### 3.3. What about the Cost of This Program?

In Switzerland, the healthcare insurance system is based on cost sharing, as the insured person pays part of the treatment's cost. This payment is made in the form of annual deductibles (called the franchise). The amount of the monthly insurance premium is then adjusted, depending on the franchise (the higher limit of the franchise is 2500 CHF per year). When the franchise exceeds, which is often the case with chronic conditions, patients pay 10% of the care costs until they reach a maximum quota of 700 CHF per year. After this quota, all care is reimbursed to the patient, including pharmaceutical services, for example, a medication adherence support fee (20.80 CHF per week) and a polymedication check fee (48.60 CHF every 6 months) [27].

### 3.4. Program Prospects

Our first prospect for this medication adherence program is to extend the research to diseases other than HIV. Studies currently in progress concern chronic dialysis, hypertension, type 2 diabetes, multiple sclerosis, and oral oncology. Cost-effectiveness analyses are also in progress.

The second prospect is to implement this program in other community pharmacies in the French-speaking part of Switzerland. The program is also in the process of being implemented for HIV patients at the Hospital of Neuchâtel (Switzerland) in collaboration with local physicians, nurses, and community pharmacies.

## 4. Conclusion

This paper thoroughly describes the experience of a well-established adherence program in Lausanne, Switzerland. It is an intervention that includes motivational interviews, electronic pill monitors, and reports, and it has interdisciplinary collaboration between all healthcare professionals. It is used with chronic patients experiencing or at risk of experiencing medication adherence issues. The fact that it is patient-centered makes it possible for the patients to develop autonomy. The program bridges research and practice, and it encourages the implementation of such a program elsewhere in the world. More articles should describe similar successful intervention programs to promote experience exchanges, comparisons, and replications in different settings.

## Supplementary Material

Published research about the medication adherence program.

## Figures and Tables

**Table 1:** Patient-level intervention according to Michie's et al. taxonomy [26].

Michie's et al. taxonomy	Intervention
Goals and planning	Set realistic goals and adjust them to build up skills, use the problem solving technique, and raise awareness on discrepancy between current behavior and goals as a motor of change

Feedback and monitoring	Electronic monitoring, empathic reinforcement, alliance through LCD display of electronic pill monitor, and ensuring continuity of care through medication adherence report

Social support	Reinforce positive practical and/or emotional support, invite significant others to attend interview, and offer the possibility to bring adherence report back home to discuss it with significant others

Shaping knowledge	Assess patient's cognitive and behavioral knowledge and needs in regard to long-term adherence, short-term and long-term side effects, fill in gaps with adequate vocabulary, and reevaluate needs over time

Natural consequences	Evaluate consequences, which are relevant to the patient (e.g., health, quality of life, and social, emotional, affective, financial, and professional consequences) and use hypotheses as a motor of potential changes (e.g., what would happen if you would take your medication on a regular basis?)

Comparison of behavior	Ask the permission for telling what other patients did in a similar situation

Associations	Associate drug intake with relevant individual daily actions, behaviors, cues, and reminders

Repetition and substitution	Plan short but repeated interviews over time, adjusted to patients' needs

Comparison of outcomes	Compare change in clinical outcomes and in adherence and set future goals

Reward and threat	Congratulate patient on achievements as small as they are; if necessary, evoke risks cautiously with patient agreement

Regulation	Detangle possible triangulation between patient and healthcare providers, listen to and regulate emotions, and, if possible, wait and see if patient is not ready to change behavior (preparation phase)

Antecedents	Evaluate adherence with past treatments as indicator

Identity	Reinforce patient positive behaviour, respect patient's rhythm and possibilities, and keep contact with patient (e.g., schedule a new interview in case of a missed appointment)

Scheduled consequences	Identify changes in clinical outcomes

Self-belief	Explore patient's past success, empower patient, and support patient in building self-confidence, self-efficacy, and motivation with treatment

Covert learning	—

**Table 2:** Time intervals in-between interviews and time per patient visit to the medication adherence program (visits *n* = 7171).

	Median	IQR
Time intervals in-between interviews [days]		
HIV	34	[15–78]
Multiple sclerosis (MP)	28	[20–42]
Oncology	49	[30–95]
Other chronic conditions	35	[19–64.5]
Total	**33**	[**15–77**]

Time needed for inclusion interviews [minutes]		
Interview	10	[5–15]
Report	25	[17–36.5]
EMs handling	20	[15–25]
Total	**60**	[**45–76.5**]

Time needed for follow-up interviews [minutes]		
Interview	10	[5–15]
Report	12	[7–20]
EMs handling	13	[9–20]
Total	**38**	[**27–50**]

*Note*. IQR = interquartile range.
