# Supplementary material for: Interdisciplinary Medication Adherence Program: The Example of a University Community Pharmacy in Switzerland
Source: Biomed Res Int. 2016 Jan 10;2015:103546. doi: 10.1155/2015/103546 (PMC4709610; doi:10.1155/2015/103546)
Supplement: Supplementary file 1 — Published research about the medication adherence program. [file 103546.f1.pdf]

Appendix 1: published research about the medication adherence program

Article	Aim of the study	Studied population and study duration	Design	Outcomes	Results
**Fallab-Stubi et al., 1998 [28]**	Evaluate the usefulness of EMs, comparing with pill count method and urine test.	Tuberculin positive patients treated with isoniazid<br><br>N = 30<br><br>Period: 6 months	Prospective study:<br>Visits planned every 4 weeks<br>+ Combined intervention (physician and pharmacist) with non-adherent patients at 3 and 6 months.	- Medication adherence	Adherence with EMs: 91.5 +- 18.6 %,<br>With pill count: 96.2 +- 15.2 %,<br>With urine test: 91.3 +- 22.2 %.<br>EMs appeared to be more reliable than the pill count and urine test, which tended to overestimate the overall adherence. Combined intervention enhanced adherence in non-adherent patients.
**Schneider et al., 1998 [29]**	Assess if on-line monitoring of medication adherence is feasible and acceptable to patients.	Patients with chronic uncomplicated diseases (diabetes, hypertension, hypercholesterolaemia)<br><br>N = 7<br><br>Period: 2 months	Open pilot study:<br>- 4-weeks running phases with EMs<br>- After 1 month: a modem connected to the pharmacy was installed on the patient's telephone line and EMs were installed on the modem every evening by the patient<br>- In the end of the study: patients completed a satisfaction questionnaire.	- Medication adherence	Adherence > 95 % : 4 patients<br>Adherence < 95 %: 3 patients but adherence increased during the on-line monitoring period.<br><br>On-line monitoring seems to be feasible and acceptable to patients.
**Schwed et al., 1999 [30]**	Determine adherence to lipid-lowering therapy, using an EMs vs. pill count.<br>Illustrate pattern of non adherence.<br>Evaluate the relationship between adherence and response to fluvastatin.	Hypercholesterolemia<br><br>N = 39<br><br>Period: 9 months	Open label study:<br>- Dietetic phase: 3 months with 3 clinical visits<br>- Active treatment phase (fluvastatin): 6 months with 3 clinical visits (at 1, 3 and 6 months).	- Medication adherence<br>- Pattern of non adherence<br>- Total cholesterol and LDL cholesterol	Lipid profile was improved during the active treatment phase but not during dietetic phase.<br>Adherence was > 80 % at each visit but decreased over time. Pill count overestimated adherence data.<br>Patterns of non adherence observed: drug holiday, missed doses, multiple daily doses, or toothbrush effect.<br>Correlation between rate of adherence and decrease in LDL cholesterol: significant with EMs but not with pill count.

**Bertholet et al., 2000 [31]**	Evaluate the effect of the use of EMs on identifying and correcting adherence problems and its ultimate effect on enhancing the clinical outcome	Hypertensive patients (BP > 140/90 mm Hg)<br><br>N = 69<br><br>Period: no information	Prospective study: Antihypertensive therapy dispensed in EMs.	- Medication adherence<br>- Blood pressure	Decrease of blood pressure (from 159/104 +/- 23/12 to 143/92 +/- 20/15 mm Hg) for the group but:<br>The use of electronic monitors had a positive effect on enhancing the therapy of antihypertensive patients.
**Burnier et al., 2001 [19]**	Evaluate the potential benefits EMs in the management of patients with resistant hypertension.	Hypertensive patients resistant to a 3-drug regimen<br><br>N = 41<br><br>Period: 4 months	Prospective study:<br>2 months follow-up with EMs. After this period, patients could continue with EMs during 2 more months and treatment was adapted if necessary	- Medication adherence<br>- Blood pressure	At 2 months: significant improvement of blood pressure. Patients with lowest adherence had higher diastolic blood pressure.<br>At 4 months (n=30/41): further significant decrease in blood pressure.
**Schneider et al., 2003 [32]**	Evaluate the efficacy of a nicotine nasal spray in smoking cessation and characterize the pattern of use of the nasal spray with a special electronic monitor to assess factors associated with cessation success or failure.	Patient referred to the smoking cessation unit from October 1996 to April 1997<br><br>N = 92<br><br>Period: 24 months	Open prospective exploratory study: patients received a nicotine nasal spray with a microchip (MDILog) for 18 months.<br>Follow-up visits were scheduled at week 2 and at 1,2, 3, 4, 6, 9, 12, 15, 18, 21 and 24 months.	- Number of abstainers at 12 and 24 months<br>- Use of the spray	First 2 weeks: abstinence was significantly associated with cessation success at 6 and 24 months.<br><br>First month: median use of 12 puffs/day in abstainers and 6 puffs/day in failures.<br>Abstainers used the spray less in the morning but more in the evening/night compared with failures.<br><br>Mean daily consumption of the nicotine nasal spray remained lower than expected in most participants, particularly in failures.
**Schneider et al., 2003 [33]**	Evaluate the benefit of an online telemonitoring of medication adherence.	Epileptic patients<br><br>N = 26 (12 in the intervention group)<br><br>Period: 8 months	Pilot randomized controlled study:<br>- Control: antiepileptic drugs in EMs<br>- Intervention: EMs combined with online telemonitoring. The pharmacist called patients if adherence problems occurred.	- Medication adherence	Percentage of days with correct dosing: > 90 % in control and intervention groups.<br><br>Number of interventions performed by pharmacists: 78 (4 patients had more than 10 interventions).<br><br>Intervention group: trend to fewer epileptic events vs. control.

**Landry et al., 2006 [34]**	Evaluate adherence to malaria chemoprophylaxis with weekly mefloquine.<br><br>Compare EMs adherence with usual care questionnaire.	Adult travellers to sub-Saharan Africa<br><br>N = 81<br><br>Period: March to April 1997	Before Travelling: oral and written information received about malaria and mefloquine + questionnaire on demographic and travel data, medical history and current medical treatment + patients received mefloquine in EMs.<br><br>After travelling: second questionnaire on protective measures, drug intake and adverse events.	- Medication adherence<br>- Demographics and Travel Characteristics<br>- Adverse events	Electronic adherence data:<br>- 75.3% took all tablets: 32.1% at the expected date, 9.9% starting late, 23.5% with intervals of ± 1 day from the right date and 9.9% took pills in a random way<br>- 24.7% missed some doses mainly after return.<br><br>Questionnaire: higher strict adherence data, some answers not reliable (21 cases with discordant results).
**Santschi et al., 2007 [35]**	Evaluate the acceptability of a new electronic drug adherence monitor (IDAS II).	Hypertensive patients treated with irbesartan<br><br>N = 24<br><br>Period: 4 months	randomised two-way cross-over study:<br>Device 1, 2 months: IDAS II<br>Device 2, 2 months: MEMS	- Patient's opinion on both devices<br>- Medication adherence<br>- Blood pressure	Both devices: reliable reminders<br>Preferences: MEMS (10/24), IDAS II (11/24), no preference (3/24)<br>MEMS: easier to use than IDAS II<br>IDAS II: better packaging than MEMS<br><br>Adherence: 99.2 % with both devices, higher regularity of drug intake timing with IDAS II.
**Santschi et al., 2008 [36]**	Investigate if having the possibility to use EMs with interdisciplinary networks improved blood pressure control.	Uncontrolled hypertensive patients<br><br>N = 68 (34 in the intervention group)<br><br>Period: 12 months	Pragmatic cluster randomised controlled study:<br>Intervention: medication adherence monitored with EMs<br>Control: usual care.	- Target office BP<140/90 mmHg	Significantly more patients reached target BP in the intervention group at 4 and 12 months vs. control group.

**Krummenacher et al., 2010 [20]**	Evaluate the feasibility of a comprehensive, interdisciplinary adherence program	HIV patients followed by the Swiss HIV Cohort Study in 2 centres: Lausanne and Basel (Switzerland)<br><br>N = 32 (21 in the intervention group)<br><br>Period: 6 months, November 2006 to July 2007	Pilot study, quasi-experimental with 2 arms:<br>- intervention: usual care + EMs + motivational interviews with a pharmacist.<br>- control: usual care + blinded EMs	- Rate of patients' acceptance to take part in the program<br>- Rate of patients' retention in intervention and control groups<br>- Adherence, implementation and persistence	Study was not feasible in Basel: the recruitment stopped because unsuccessful inclusion.<br>But feasible in Lausanne:<br>- Inclusion rate intervention vs. control: 84% vs 52 %<br>- Retention rate: 91% vs 82%<br>- Adherence: 93% vs 87% but adherence decreased more quickly in the control group<br>- Implementation: 97% vs 95% and decrease with time in both groups<br>- Persistence: 97% vs 81%.
**Krummenacher et al., 2011 [21]**	Analyze the interdisciplinary HIV-adherence program	HIV patients referred to the program<br><br>N = 104<br><br>Period: August 2004 to April 2008	Retrospective cohort study	- Socio-demographic data<br>- Reasons for inclusion<br>- Adherence rate<br>- Clinical data (VL and CD4 cells)<br>- Description of the pharmacy visits<br>- Reasons for antiretroviral treatment adjustments<br>- Reasons for interruption of the program.	59% women, 42% black ethnicity and median age = 39. 77% patients were ART-experienced and 59% had a protease inhibitor.<br>Retention rate in the program: 92%<br>Reasons for inclusion: naive patients, non adherence or suboptimal clinical outcomes<br>Adherence: 87% persistence and 88% implementation. Number of undetectable patients increased during the study.<br>Median time of interview: 35 minutes<br>ART Adjustments: 45% (41% treatment simplification, 20% adverse events, 20% clinical issues)

**Krummenacher et al., 2012 [22]**	Describe demographic and clinical characteristics of HIV patients referred to the adherence program in comparison to the entire HIV population.	HIV patients referred to the adherence program N = 73<br><br>HIV patients followed in the same infection disease department N = 494<br><br>Period: August 2007 to November 2007	Retrospective descriptive cross-sectional study	- Socio-demographic data<br>- Clinical data: VL and CD4 cells	Subjects referred to the adherence program differed from the entire HIV population:<br>- Women more often included (66%)<br>- Protease inhibitor more often prescribed than NNRTI<br>- Lower CD4 cell count<br>- Patients more often under salvage therapy.
**Gertsch et al., 2013 [24]**	Describe adherence of pregnancy and postpartum HIV women referred to the adherence program.	Pregnant HIV women referred to the adherence program<br><br>N = 25<br><br>Period: 2004 to 2012	Exploratory, retrospective study	- Socio-demographic data<br>- Adherence data	68% black ethnicity, 44% naive patients, median age= 29<br>96% has a protease inhibitor and NRTIs<br>Women who stopped the program:<br>- 24% during pregnancy, 12% at childbirth and 16% during postpartum period<br>- 62% kept on ART, 3 stopped ART after childbirth and 2 stopped without physician's agreement<br>Unattended visits: increased from 17% during pregnancy to 38% during postpartum<br>Probability of correct ART intake: 97% during pregnancy, 93% at childbirth and increased again during postpartum.
**Krummenacher et al., 2014 [23]**	Identify barriers and facilitators encountered by HIV patients with suboptimal medication adherence.	HIV patients referred to the adherence program<br><br>N = 17<br><br>Period: August 2004 to May 2008	Retrospective, qualitative, thematic content analysis of pharmacists' notes during interviews conducted within the adherence program.	- Barriers and facilitators	Three main categories of barriers/facilitators:<br>- Cognitive, emotional and motivational<br>- Environmental, organisational and social<br>- Treatment and disease.

References

[1] World Health Organization, *Adherence to Long-Term Therapies—Evidence for Action*, 2003, http://www.who.int/chp/knowledge/publications/adherence_report/en/.

[2] B. Vrijens, S. De Geest, D. A. Hughes et al., "A new taxonomy for describing and defining adherence to medications," *British Journal of Clinical Pharmacology*, vol. 73, no. 5, pp. 691–705, 2012.

[3] C. Ortego, T. B. Huedo-Medina, J. Llorca et al., "Adherence to highly active antiretroviral therapy (HAART): a meta-analysis," *AIDS and Behavior*, vol. 15, no. 7, pp. 1381–1396, 2011.

[4] L. A. Jeanneret, F. Lüthi, M. P. Schneider, S. Troxler, and O. Bugnon, "Adhésion thérapeutique aux traitements oncologiques oraux et prise en charge interdisciplinaire," *Revue Médicale Suisse*, vol. 7, no. 296, pp. 1154–1160, 2011.

[5] R. Nieuwlaat, W. Wilczynski, T. Navarro et al., "Interventions for enhancing medication adherence," *The Cochrane Database of Systematic Reviews*, vol. 11, Article ID CD000011, 2014.

[6] IMS Institute for Healthcare Informatics, "Advancing the responsible use of medicines—applying levers for change—summary," p. 1–24, 2012, http://www.imshealth.com/portal/site/imshealth/menuitem.762a961826aad98f53c753c71ad8c22a/?vgnextoid=faf9ee0a8e631410VgnVCM10000076192ca2RCRD&vgnextchannel=736de5fda6370410VgnVCM10000076192ca2RCRD&vgnextfmt=default.

[7] O. Bugnon and M. Buchmann, "Pharmacie d'officine et médecine interne générale à la croisée des mêmes chemins: des opportunités à saisir," *Revue Médicale Suisse*, vol. 8, no. 364, pp. 2287–2291, 2012.

[8] M.-P. Schneider and P. Aslanio, "Adherence policy, education and practice—an international perspective," *Pharmacy Practice*, vol. 8, no. 4, pp. 209–212, 2010.

[9] D. Drotar, "Strategies of adherence promotion in the management of pediatric chronic conditions," *Journal of Developmental and Behavioral Pediatrics*, vol. 34, no. 9, pp. 716–729, 2013.

[10] Association Pharmaceutique Belge (APB), "Entretien d'accompagnement de nouvelle médication—L'ENM: un train à ne pas manquer," *Annales Pharmaceutiques Belges*, vol. 9, 2013.

[11] K. M. Wells, T. Thornley, M. J. Boyd, and H. F. Boardman, "Views and experiences of community pharmacists and superintendent pharmacists regarding the New Medicine Service in England prior to implementation," *Research in Social and Administrative Pharmacy*, vol. 10, no. 1, pp. 58–71, 2014.

[12] NICE, "Guidelines CG76," 2009, http://www.nice.org.uk/guidance/cg76/resources.

[13] J. Demonceau, T. Ruppar, P. Kristanto et al., "Identification and assessment of adherence-enhancing interventions in studies assessing medication adherence through electronically compiled drug dosing histories: a systematic literature review and meta-analysis," *Drugs*, vol. 73, no. 6, pp. 545–562, 2013.

[14] http://www.medamigo.com.

[15] https://www.sispha.com/.

[16] J. D. Fisher, W. A. Fisher, K. R. Amico, and J. J. Harman, "An information-motivation-behavioral skills model of adherence to antiretroviral therapy," *Health Psychology*, vol. 25, no. 4, pp. 462–473, 2006.

[17] J.-B. Daeppen, C. Fortini, N. Bertholet et al., "Training medical students to conduct motivational interviewing: a randomized controlled trial," *Patient Education and Counseling*, vol. 87, no. 3, pp. 313–318, 2012.

[18] O. Bugnon, D. Hugentobler-Hampaï, J. Berger, and M. P. Schneider, "New roles for community pharmacists in modern health care systems: a challenge for pharmacy education and research," *Chimia*, vol. 66, no. 5, pp. 304–307, 2012.

[19] M. Burnier, M. P. Schneider, A. Chioléro, C. L. F. Stubi, and H. R. Brunner, "Electronic compliance monitoring in resistant hypertension: the basis for rational therapeutic decisions," *Journal of Hypertension*, vol. 19, no. 2, pp. 335–341, 2001.

[20] I. Krummenacher, M. Cavassini, O. Bugnon, R. Spirig, and M. P. Schneider, "Antiretroviral adherence program in HIV patients: a feasibility study in the Swiss HIV Cohort Study," *Pharmacy World and Science*, vol. 32, no. 6, pp. 776–786, 2010.

[21] I. Krummenacher, M. Cavassini, O. Bugnon, and M. P. Schneider, "An interdisciplinary HIV-adherence program combining motivational interviewing and electronic antiretroviral drug monitoring," *AIDS Care*, vol. 23, no. 5, pp. 550–561, 2011.

[22] I. Krummenacher, M. Cavassini, O. Bugnon, and M. P. Schneider, "Characteristics of HIV patients referred to a medication adherence program in Switzerland," *International Journal of Clinical Pharmacy*, vol. 34, no. 3, pp. 426–431, 2012.

[23] I. Krummenacher, B. Spencer, S. Du Pasquier, O. Bugnon, M. Cavassini, and M. P. Schneider, "Qualitative analysis of barriers and facilitators encountered by HIV patients in an ART adherence programme," *International Journal of Clinical Pharmacy*, vol. 36, no. 4, pp. 716–724, 2014.

[24] A. Gertsch, O. Michel, I. Locatelli et al., "Adherence to antiretroviral treatment decreases during postpartum compared to pregnancy: a longitudinal electronic monitoring study," *AIDS Patient Care and STDs*, vol. 27, no. 4, pp. 208–210, 2013.

[25] WHPA, "World's health professions call for new emphasis on working together," 2013, http://www.whpa.org/WHPA_Statement_collaborative_practice.pdf.

[26] S. Michie, M. Richardson, M. Johnston et al., "The behavior change technique taxonomy (v1) of 93 hierarchically clustered techniques: building an international consensus for the reporting of behavior change interventions," *Annals of Behavioral Medicine*, vol. 46, no. 1, pp. 81–95, 2013.

[27] E. Guignard and O. Bugnon, "Pharmaceutical care in community pharmacies: practice and research in Switzerland," *The Annals of Pharmacotherapy*, vol. 40, no. 3, pp. 512–517, 2006.

[28] C.-L. Fallab-Stubi, J. P. Zellweger, A. Sauty, C. Uldry, D. Iorillo, and M. Burnier, "Electronic monitoring of adherence to treatment in the preventive chemotherapy of tuberculosis," *International Journal of Tuberculosis and Lung Disease*, vol. 2, no. 7, pp. 525–530, 1998.

[29] M.-P. Schneider and M. Burnier, "On-line home monitoring of drug compliance: is it feasible?" *European Journal of Clinical Pharmacology*, vol. 54, no. 6, pp. 489–490, 1998.

[30] A. Schwed, C.-L. Fallab, M. Burnier et al., "Electronic monitoring of compliance to lipid-lowering therapy in clinical practice," *Journal of Clinical Pharmacology*, vol. 39, no. 4, pp. 402–409, 1999.

[31] N. Bertholet, B. Favrat, C. L. Fallab-Stubi, H. R. Brunner, and M. Burnier, "Why objective monitoring of compliance is important in the management of hypertension," *The Journal of Clinical Hypertension*, vol. 2, no. 4, pp. 258–262, 2000.

[32] M.-P. Schneider, G. van Melle, C. Uldry et al., "Electronic monitoring of long-term use of the nicotine nasal spray and predictors of success in a smoking cessation program," *Nicotine and Tobacco Research*, vol. 5, no. 5, pp. 719–727, 2003.

[33] M. P. Schneider, P. A. Despland, T. Buclin, and M. Burnier, "Evaluation of online telemonitoring of drug adherence: a pilot

randomised, controlled study in patients with epilepsy," *The Journal on Information Technology in Healthcare*, vol. 1, no. 6, pp. 419–435, 2003.

[34] P. Landry, D. Iorillo, R. Darioli, M. Burnier, and B. Genton, "Do travelers really take their mefloquine malaria chemoprophylaxis? Estimation of adherence by an electronic pillbox," *Journal of Travel Medicine*, vol. 13, no. 1, pp. 8–14, 2006.

[35] V. Santschi, G. Wuerzner, M.-P. Schneider, O. Bugnon, and M. Burnier, "Clinical evaluation of IDAS II, a new electronic device enabling drug adherence monitoring," *European Journal of Clinical Pharmacology*, vol. 63, no. 12, pp. 1179–1184, 2007.

[36] V. Santschi, N. Rodondi, O. Bugnon, and M. Burnier, "Impact of electronic monitoring of drug adherence on blood pressure control in primary care: a cluster 12-month randomised controlled study," *European Journal of Internal Medicine*, vol. 19, no. 6, pp. 427–434, 2008.
